# A peculiar case report of extraovarian Brenner tumor arising in the omentum

**DOI:** 10.1186/s12957-017-1135-2

**Published:** 2017-03-28

**Authors:** Chung Su Hwang, Chang Hun Lee, So Jeong Lee, Young Geum Kim, Ahrong Kim, Do Youn Park, Hyun Jeong Kang, Dong Hoon Shin

**Affiliations:** 1 Department of Pathology, Pusan National University Yangsan Hospital, Yangsan, Republic of Korea; 2 Department of Pathology and Medical Research Institute, Pusan National University Hospital, Busan, Republic of Korea; 3 Department of Pathology, Munhwa Hospital, Busan, Republic of Korea

**Keywords:** Brenner tumor, Extraovarian, Omentum

## Abstract

**Background:**

Brenner tumors almost always develop in the ovary. Exceptionally, extraovarian Brenner tumors have been reported in the lower abdomen or pelvic organs. Here, we introduce a peculiar case of an extraovarian Brenner tumor arising in the omentum.

**Case presentation:**

A 43-year-old woman presented with a palpable abdominal mass. Computed tomography (CT) scan revealed a 9.0-cm solid mass in the omentum. The tumor was not associated with pelvic structures, including the ovaries. It was excised under the clinical impression of an extragastrointestinal stromal tumor or neurogenic tumor. Grossly, the mass was a well-circumscribed solid tumor, with yellow-tan cut surface and minute cystic spaces. Microscopically, the tumor showed well-defined epithelial nests with variable cystic changes embedded in an abundant fibrous stroma. The cells within the nests were reminiscent of benign urothelial cells in that they had oval, frequently grooved nuclei. The epithelial cells focally showed a gradual transition into the surrounding stromal cells with short spindled features. The urothelium-like cells were positive for pancytokeratin, WT-1, p63, CK7, uroplakin-III, and GATA-3 but were negative for CD34, CD10, CK20, c-KIT, DOG-1, PAX-8, and calretinin. Morphological and immunohistochemical features of the tumor were the same as an ovarian Brenner tumor, and so it was diagnosed as an extraovarian Brenner tumor.

**Conclusions:**

Although the location of the tumor was very unusual, we could diagnose the tumor as an extraovarian Brenner tumor on the basis of the histologic and immunohistochemical findings. This is the first case of extraovarian Brenner tumor arising in the omentum near the stomach ever reported in the English literature.

## Background

The Brenner tumor was first described by Fritz Brenner in 1907 [1], who postulated that it was derived from the granulosa cells of the ovarian follicles. Their histology consists of solid or cystic urothelium-like epithelial cell nests with surrounding fibrous stroma. Interestingly, Brenner tumors can develop in extraovarian sites and have the same histologic features as those of ovarian Brenner tumors. Extraovarian Brenner tumors are exceptionally rare. Sixteen cases of extraovarian Brenner tumors have been reported to date. They have been described in the lower abdomen or pelvic cavity such as the broad ligament and vagina, and in the testicular and paratesticular area [2, 3]. Here, we report a peculiar case of extraovarian Brenner tumor arising in the omentum that has not so far been reported in the English literature. The clinicopathological features of this tumor are presented herein with a brief review of the possible histogenesis and differential diagnosis of Brenner tumors.

## Case presentation

### Clinical summary

A 43-year-old woman visited the Department of Obstetrics and Gynecology because of fluid collection in her cul-de-sac that was incidentally found 6 months ago at a routine health check. She did not complain of any other symptom except for dull abdominal discomfort. During physical examination, a mass-like hardness could be palpated on the level above the umbilicus. Routine blood analysis was within normal limits.

A computed tomography (CT) scan of the abdomen was performed, and it revealed a 9.0-cm enhancing mass in the omentum at the right upper quadrant of the abdomen. The mass showed a relatively demarcated margin and some minute cystic changes. The radiologist thought the mass was a gastrointestinal stromal tumor (GIST) or neurogenic tumor. Additionally, there was a noted 2.5-cm corpus luteal cyst in the left ovary but otherwise there were no other abnormalities in the abdominal and pelvic cavities. The clinicians decided to excise the mass due to its huge size and to accurately diagnose the tumor.

In the operative field, the mass was located around the distal antrum along the greater curvature of the stomach but was easily separated from the stomach wall itself. On the other hand, it was densely adhered to the omentum and mesocolon. The tumor was far away from the reproductive organs, such as the uterus and both ovaries, and urinary structures.

### Pathological findings

**Fig. 1:**
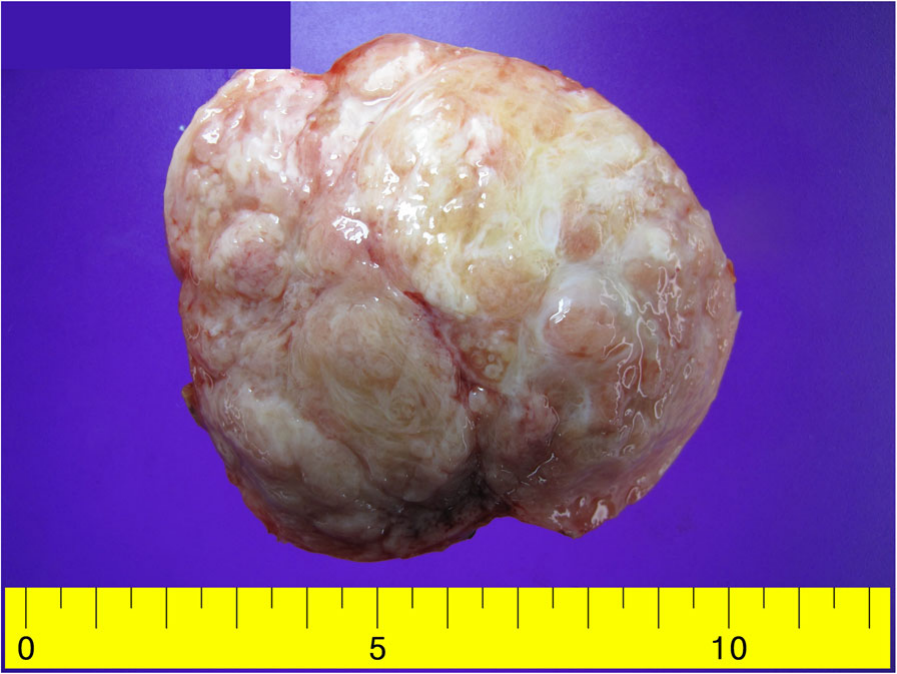
The gross appearance of the tumor. The cut surface is *yellow pink* and has lobulated features. The tumor also shows focal cystic changes

On gross pathological examination, the excised mass measured 9.5 × 8.0 × 7.5 cm. On sectioning, the cut surface of the mass was yellow pink and showed a solid lobular portion and focal small cystic areas. Its margin was well demarcated from the attached omental fat tissue (Fig. 1).

**Fig. 2:**
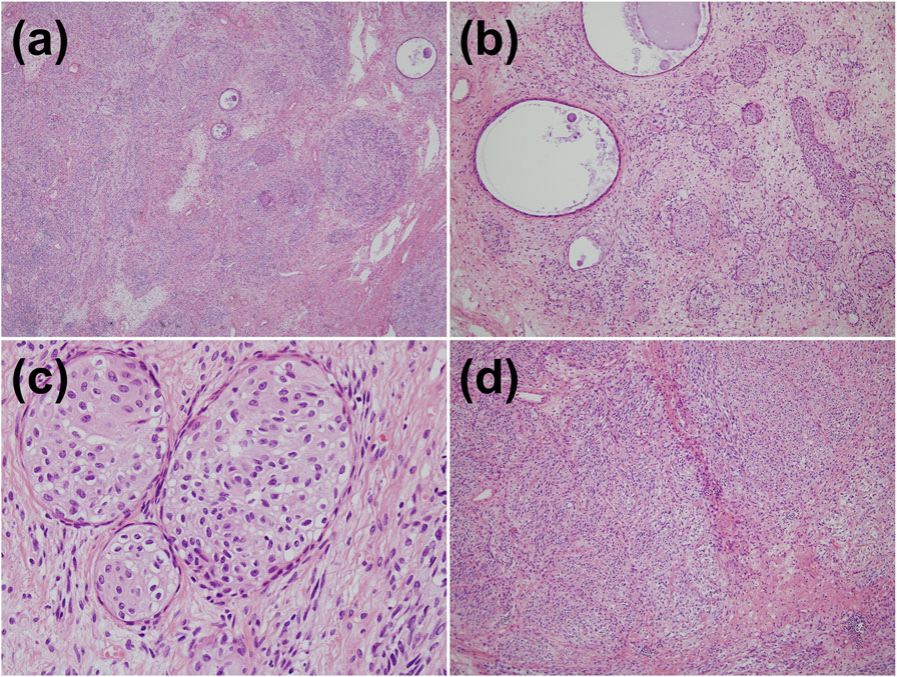
The microscopic findings of the tumor. (**a**) The tumor consists of the cellular components and hyalinized fibrous stroma. The cellular components are composed of well-defined epithelial nests of round or polygonal cells and ill-defined nodular clusters of the epithelial cells with short spindled features. (**b**) Well-defined nests of urothelium-like cells are scattered throughout the tumor. Large cystic changes within the cell nests are often identified. (**c**) The epithelial cells within well-defined nests show elongated nuclei with a nuclear groove and a perinuclear halo. The cells also have clear to pale eosinophilic cytoplasm. (**d**) The epithelial cells with short spindled features form numerous ill-demarcated lobular clusters. Abundant hyalinized fibrous stroma is present around the well-defined nests and lobular lesions

Microscopically, the tumor showed two histologic components. The first cellular part consisted of well-defined nests of round or polygonal epithelial cells and numerous ill-defined lobular islands of epithelial cells with short spindled features (Fig. 2a). The nests of polygonal epithelial cells were intimately related to the surrounding epithelial cells with short spindled features (Fig. 2b). In the well-defined nests, the epithelial cells had elongated nuclei and rather pale eosinophilic cytoplasm. Characteristically, many cells showed a longitudinal groove in the nuclei and frequent perinuclear haloes (Fig. 2c). Their cytologic features were similar to that of normal urothelial cells. Some nests showed cystic changes of varying degrees (Fig. 2b). The epithelial cells with short spindled features were arranged into ill-defined lobules or scattered singly and showed poorly defined cytoplasmic outlines (Fig. 2d). In the nuclei, the nuclear chromatin was fine and the nucleoli were small or inconspicuous. The cells also displayed occasional nuclear grooves. Neither cytologic atypia nor mitotic activity was noted in both types of cells. The second component was densely hyalinized fibrous stroma occupying areasbetween the cellular parts. The microscopic features of this tumor as a whole closely resembled an ordinary ovarian Brenner tumor.

### Immunohistochemistry

We performed immunohistochemistry on a Leica Bond-Max automatic slide immunostainer (Leica Biosystems Melbourne Pty., Ltd. VIC, Australia) using a standard protocol. The list of antibodies used is as follows: calretinin (Novocastra, diluted 1:200), CK7 (NeoMarkers, 1:400), CK20 (Novocastra, 1: 100), pancytokeratin (Novocastra, 1:100), CD10 (Novocastra, 1:150), CD34 (DAKO, 1:400), DOG-1 (Cell Marque, 1:250), c-KIT (DAKO, 1:300), p63 (Novocastra, 1:100), PAX-8 (Cell Marque, 1:200), SMA (DAKO, 1:400), vimentin (Zymed, 1:200), WT-1 (DAKO, 1:100), uroplakin-III (Cell Marque, 1:50), and GATA-3 (Cell Marque, 1:100).

**Fig. 3:**
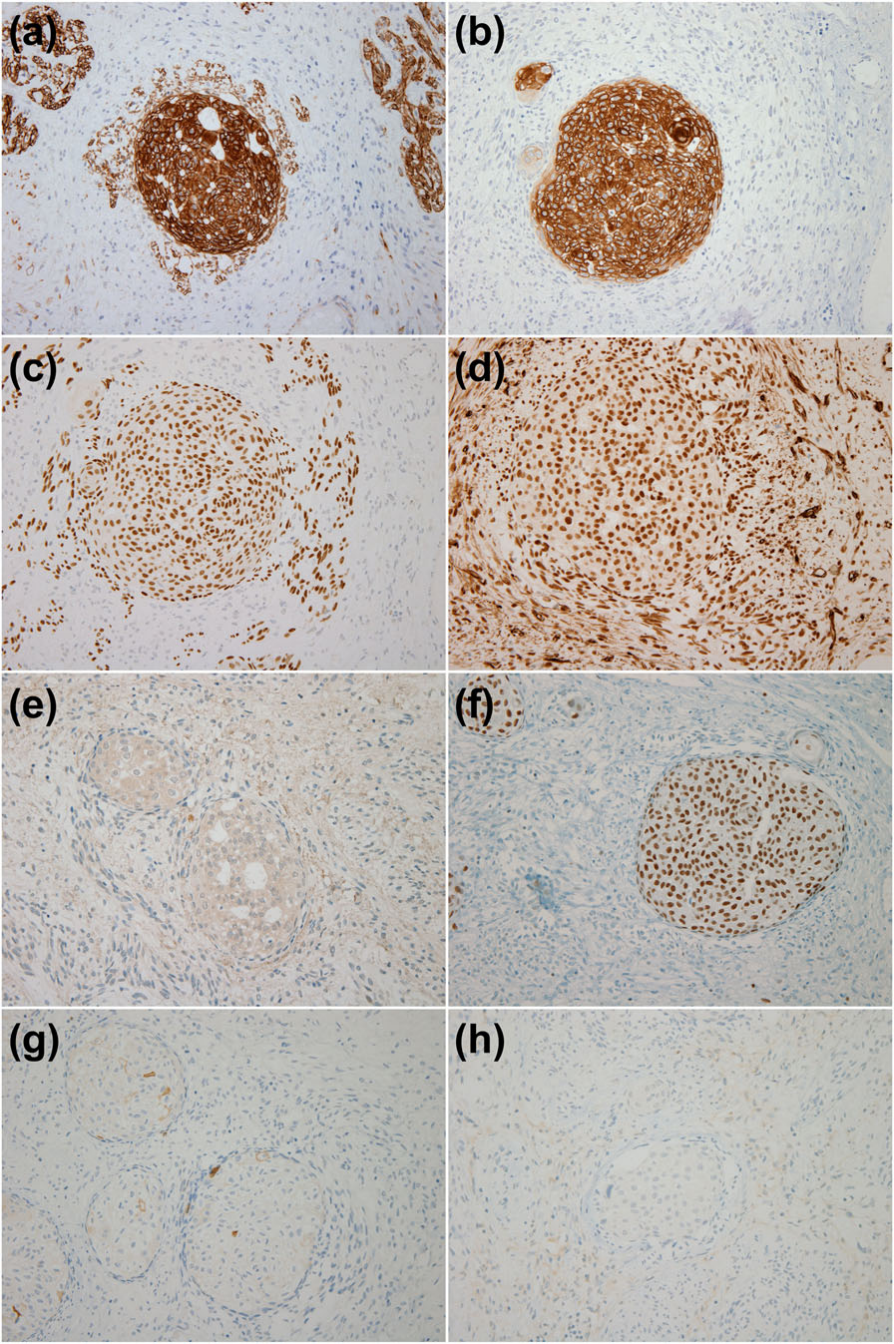
The results of immunohistochemical staining. (**a**) Both urothelium-like cell nests and ill-defined lobular clusters of spindled epithelial cells are positive for pancytokeratin. (**b**) The urothelium-like cell nests are positive for CK7, but the spindled epithelial cells are negative. Both urothelium-like cell nests and spindled epithelial cells are also positive in the nuclei for (**c**) p63 and (**d**) WT-1. Only the well-defined urothelium-like cell nests are diffuselt weakly positive in the cytoplasm for (**e**) uroplakin-III and diffusely strongly positive in the nuclei for (**f**) GATA-3. All epithelial cells and stroma are negative for (**g**) c-KIT and (**h**) DOG-1

The cellular parts, including the urothelium-like cells and spindled epithelial cells were positive for pancytokeratin, p63, and WT-1 but negative for CD34, CD10, CK20, calretinin, c-KIT, DOG-1, and PAX-8. The urothelium-like cells were positive for CK7 but the spindled epithelial cells were negative. The cytoplasm of the urothelium-like cells was weakly to moderately positive for uroplakin-III, and their nuclei were diffusely strongly positive for GATA-3. The immunoprofiles of the urothelial-type cell nests in this tumor were the same as those of ovarian Brenner tumors. The spindled epithelial cells were negative for both uroplakin-III and GATA-3. The stromal cells only showed focal positivity for SMA (Fig. 3).

### Mutation analysis of *c-KIT*

Using a representative paraffin block of the tumor, we performed mutation analyses for exons 9, 11, 13, and 17 of the *c-KIT* gene by polymerase chain reaction (GeneAmp PCR System 2700, USA) using a direct sequencing method (Applied Biosystems 3500 Genetic Analyzer, USA). The results of the *c-KIT* gene mutation analyses were negative.

### Follow-up

After surgery, the patient had recovered well and showed no recurrence during the 17-month follow-up.

## Discussion

Extraovarian Brenner tumor was first reported by Robinson in 1950 [4]. Ruthy Shco-Levy et al. reported an extraovarian Brenner tumor that developed in the right distal vaginal wall and investigated the prior reported cases of extraovarian Brenner tumor in the English literature [2]. They found that 14 cases of extraovarian Brenner tumor have been reported with 5 in the vagina, 4 in the broad ligament, 3 in the testis, 1 in the uterus, and 1 in the paratesticular area. All of the extraovarian Brenner tumors reported so far were characteristically found near the reproductive organs. However, it is interesting that in our case an extraovarian Brenner tumor was found in the omentum far from the reproductive organs.

Reported extraovarian Brenner tumors showed very similar histologic findings. The tumors were composed of epithelial cell nests resembling benign urothelium and hyalinized stroma surrounding the urothelial-type cell nests [2, 5–8]. Some epithelial cell nests showed squamous metaplasia, glandular structures, or cystic changes [2, 8]. In the present case, we found that benign-looking epithelial cells with short spindled features formed ill-defined lobules around or away from the urothelial-type epithelial nests. The short spindled epithelial cells occasionally showed a longitudinal nuclear groove and were positive for pancytokeratin, p63, and WT-1, as were the urothelial-type cell nests. Therefore, we assume that the short spindled epithelial cells are less differentiated but would show a differentiation lineage pattern towards the urothelial-type epithelial cells.

From the light and electron microscopic features, ovarian Brenner tumors are most likely derived from coelomic ovarian surface epithelium or cortical inclusions that have undergone urothelial or transitional metaplasia and have then progressively grown into the substance of the ovary [9]. However, the presence of the tumor in men and at sites far away from the ovary indicates that this neoplasm is not invariably of ovarian origin. The concept of this neoplasm originating from ovarian coelomic epithelium via a process of Wolffian differentiation has also been supported by serial reconstruction studies [10]. When taking into consideration that the abdominal and pelvic peritoneum are embryologically derived from the coelomic epithelium, we conclude that Brenner tumors can also develop in extraovarian sites such as in the pelvic structures and omentum. In the present case, both urothelial-type and spindled epithelial cells showed the immunohistochemical expression of WT-1 and pancytokeratin, which can be commonly seen in peritoneal mesothelial cells. Together with a mixed morphologic pattern of urothelial-type and spindled epithelial cells in cellular areas, these immunophenotypic characteristics suggest that this tumor might be derived from urothelial metaplasia of coelomic epithelium-derived peritoneum.

Although the histologic features of the tumor were very typical of ovarian Brenner tumor, other mesenchymal or biphasic tumors should be excluded because of the unusual intra-abdominal location from which this tumor arose. First, extragastrointestinal stromal tumor (EGIST) that arises in the omentum needs to be ruled out. In particular, epithelioid-type EGISTs can show similar histologic features with those of extraovarian Brenner tumor. Epithelioid-type EGISTs are composed of cells with abundant eosinophilic or clear cytoplasm. The stroma can show hyalinization and myxoid changes. The tumors may show multinucleated giant cells, binucleated cells, or cells with bizarre nuclei. In the present case, we did not observe any of these types of cells. In addition, the tumor was negative for c-KIT and DOG-1 immunostaining and for a *c-KIT* gene mutation study.

Miettinen et al. reported three cases of gastric epitheliomesenchymal biphasic tumor and proposed the term “gastroblastoma” [11]. Only six cases of gastroblastoma have been reported [11–14]. Gastroblastoma consists of uniform spindled cells with blunt-ended nuclei and epithelioid cell clusters. The clusters of epithelioid cells can show primitive features or form glands, sheets, cords, or rosette-like structures. Wey et al. observed that most of the nuclei of the epithelial cells were grooved [13]. At a glance, the histologic features of gastroblastoma might be similar to those of the present case. However, all reported gastroblastomas were quite different immunohistochemically from the present case. Ma et al. investigated the characteristics of the immunohistochemical profile of all reported gastroblastomas, including their case [14]. Gastroblastomas showed no positivity for p63 immunostaining in epithelial cell components, whereas the epithelial components of the present case showed diffuse strong positivity for p63. Topographically, all gastroblastomas are known to arise from the gastric wall. The present case definitely developed from the omentum and was located far away from the stomach.

In addition, malignant tumors such as epithelioid synovial sarcoma, carcinosarcoma, and metastatic urothelial carcinoma should also be excluded. The present case showed neither mitotic activity nor recognizable cellular atypia. The patient did not have a history of malignancy including urothelial carcinoma. Therefore, we could easily exclude malignant tumors.

## Conclusions

We reported the first case of an extraovarian Brenner tumor arising from the omentum near the stomach. The histologic and immunohistochemical findings of the tumor were very similar to those of an ovarian Brenner tumor, and therefore, we diagnosed this unusual tumor as an extraovarian Brenner tumor. Meticulous recognition of the morphologic features of this type of tumor is necessary to avoid diagnostic confusion with other intra-abdominal biphasic tumors. We postulate that this tumor might have directly originated from urothelial metaplasia of coelomic epithelium-derived peritoneum.

## Abbreviations

CK20: Cytokeratin 20
CK7: Cytokeratin 7
DOG-1: Discovered on gastrointestinal stromal tumors-1
GATA-3: GATA binding protein 3
GIST: Gastrointestinal stromal tumor
PAX8: Paired box gene 8
SMA: α-Smooth muscle actin
WT-1: Wilms’ tumor 1 protein
